# The Ethanol Extract of *Fructus trichosanthis* Promotes Fetal Hemoglobin Production via p38 MAPK Activation and ERK Inactivation in K562 Cells

**DOI:** 10.1093/ecam/neq022

**Published:** 2011-08-11

**Authors:** Hui Li, Chun Hay Ko, Suk Ying Tsang, Ping Chung Leung, Ming Chui Fung, Kwok Pui Fung

**Affiliations:** ^1^Institute of Chinese Medicine, The Chinese University of Hong Kong, Hong Kong, China; ^2^State Key Laboratory of Photochemistry and Plant Resources in West China, The Chinese University of Hong Kong, Hong Kong, China; ^3^School of Life Sciences, The Chinese University of Hong Kong, Hong Kong, China; ^4^School of Biomedical Sciences, The Chinese University of Hong Kong, Hong Kong, China

## Abstract

Pharmacological stimulation of fetal hemoglobin (HbF) expression may be a promising approach for the treatment of beta-thalassemia. In this study, the effects of *Fructus trichosanthis* (FT) were investigated in human erythroleukemic K562 cells for their gamma-globin mRNA and HbF-induction activities. The role of signaling pathways, including extracellular regulated protein kinase (ERK) and p38 mitogen-activated protein kinase (MAPK), was also investigated. It was found that the ethanol extract of FT significantly increased gamma-globin mRNA and HbF levels, determined by real-time reverse transcription polymerase chain reaction and enzyme linked immunosorbent assay, respectively, in dose- and time-dependent manner. Total Hb (THb) levels were also elevated in the concentrations without cytotoxicity (<80 **μ**g mL^−1^). Pre-treatment with p38 MAPK inhibitor SB203580 blocked the stimulatory effects of FT extract in total and HbF induction. In contrast, no change in HbF was observed when treated with ERK inhibitor PD98059. Furthermore, FT ethanol extract activated p38 MAPK and inhibited ERK signaling pathways in K562 cells, as revealed in western blotting analysis. In addition, SB203580 significantly abolished p38 MAPK activation when the cells were treated with FT. In summary, the ethanol extract of FT was found to be a potent inducer of HbF synthesis in K562 cells. The present data delineated the role of ERK and p38 MAPK signaling as molecular targets for pharmacologic stimulation of HbF production upon FT treatment.

## 1. Introduction

*β*-Thalassemia is an inherited autosomal recessive disease commonly found in the Mediterranean, southeast Asia and southern China [1, 2]. It is caused by mutations in the beta-globin gene on chromosome 11p15.5. Patients suffer great morbidity and mortality, including transfusion-dependent anemia with concomitant iron overload, hepatosplenomegaly and ineffective erythropoiesis [3]. Increased fetal hemoglobin (HbF) production can compensate for the shortfall of adult Hb (HbA) synthesis in *β*-thalassemia patients [4]. Some chemotherapeutic agents, such as hydroxyurea (HU) and 5-azacytidine have been used to enhance HbF production [5, 6], but myelotoxicity, fear of long-term carcinogenesis and only modest responses to treatment have limited the clinical usefulness of these agents [7, 8]. In view of the lack of good therapeutic agents for this disease, we established a rational strategy on discovering new interventions to reactivate HbF production in Chinese herbal medicines by using human erythroleukemia cell line K562 as screening platform. Our pilot studies showed that the ethanol extract of *Fructus trichosanthis* (FT) could induce HbF production *in vitro*.

FT, the fruit of *Trichosanthes kirilowii* MAXIM, is one of the most frequently used Chinese herbs. According to the theory of Chinese medicine, FT is useful in treating cough due to phlegm-heat and treating the accumulation of cold-phlegm [9]. In addition, FT has been used in the treatment of diabetes, bronchial diseases, breast cancer, leukemia and Ehrlich's ascitic carcinoma in various folk remedies. However, scientific evidence on its biological activities is very limited. Only one well-documented study demonstrated that the methylene chloride fraction of FT inhibited the proliferation and induced apoptosis in human leukemic U937 cells *in vitro* [10]. Human erythroleukemia cell line K562 has been previously shown to be Philadelphia-chromosome positive. The expression levels of both embryonic Hb (Hb Gower and Hb Portland) and HbF is markedly increased after exposure to hemin [11]. This cell line appears to be committed along the embryo-fetal erythroid differentiation pathway and produces Hb independently of terminal differentiation [12]. Thus, it has been used extensively as a model system for the investigation of human globin gene regulation and HbF-inducer screening [13].

In order to determine the HbF-inducing effect of FT, the present study was designed to investigate the effect of FT on K562 erythroid differentiation. We found that the ethanol extract of FT promoted gamma-globin (*γ*-globin) mRNA expression and HbF production in K562 cell cultures. The inducing effect appeared to be due to the activation of p38 mitogen-activated protein kinase (MAPK) signaling pathway and the inactivation of extracellular regulated protein kinase (ERK) signaling pathway.

## 2. Methods

### 2.1. Plant Extraction

The raw materials of FT were purchased from herbal pharmaceutical company in Hong Kong and authenticated in accordance to Chinese Pharmacopeia [14]. The reference herbs were purchased from the National Institute for the Control of Pharmaceutical and Biological Products in China. The authenticated voucher specimens were deposited in the Institute of Chinese Medicine, The Chinese University of Hong Kong. For ethanol extract preparation, 500 g of dried FT was extracted by 2 l of 95% ethanol at 60°C for 24 h. This extraction step was repeated twice. After simple percolation, the collected ethanol solution was rotary evaporated at 65°C until the inner matters become viscous semi-solid. The viscous content of the extract was precipitated out by adding 2.5 volume of 40% ethanol and discarded. The ethanol content of the solution was then removed by rotary evaporation and lyophilized by freeze dryer. The powdered form of the extract was stored in a desiccator at room temperature until use.

### 2.2. K562 Cell Culture and Treatment Protocol

Human erythroleukemic K562 cells were obtained from the American Type Culture Collection. K562 cells were cultured in RPMI-1640 medium (supplemented with 10% fetal bovine serum and 1% penicillin-streptomycin) under a humidified atmosphere with 5% CO_2_ at 37°C. The cells were then subcultured at 1 × 10^4^ cells mL^−1^ when they reached semi-confluence.

FT ethanol (FT-EtOH) extract was reconstituted in dimethyl sulfoxide (DMSO) for the cell culture. The concentration of DMSO was adjusted <0.1% of final concentration of culture medium which causes no cytotoxic effect to the cells. Cells were treated with FT-EtOH extract and a known positive control HU at various concentrations. In the study of cytotoxicity, K562 cells were seeded in 96-well plates at 4 × 10^3^ cells/well in RPMI and treated with drugs for 48 h at 37°C. For total Hb (THb) and HbF assays, the cells were seeded in 96-well plates at 4 × 10^3^ cells/well and the measurements were taken daily throughout 6 days of treatment. In order to elucidate the underlying signaling pathways, inhibitors of ERK—PD98059 (25 *μ*M)—and p38 MAPK—SB203580 (10 *μ*M)—were added on K562 cultures prior to FT treatment, followed by the THb and HbF quantifications, as well as western blotting analysis. The concentration of FT-EtOH extract was optimized at 20 *μ*g mL^−1^ for mechanistic studies in our pilot experiments.

### 2.3. Cytotoxicity Assay

The cytotoxic effect of FT extract was determined using 1-(4,5-dimethylthiazol-2-yl)-(3,5)-diphenyl-formazan (MTT) colorimetric assay. In brief, after 48 h of drug treatment, MTT solution (5 mg mL^−1^ in phosphate buffered saline) was added to each well and incubated at 37°C for 3 h. The blue formazan crystals that resulted from the reduction of MTT by living cells were dissolved in DMSO and the absorbance was measured at 540 nm. Cell survival was determined as a percentage of viable cells in treated cells versus no drug-treated control group.

### 2.4. THb Assay

The relative THb production of K562 cells was determined by 3,3,5,5-tetramethylbenzidine (TMB) assay, which is based on the peroxidase-like activity of heme portion of Hb. At each timed interval, the cells of each treatment group were reacted with 5% TMB (w/v) solution at 37°C for 10 min in darkness, followed by reaction termination by adding 2 M H_2_SO_4_. The number of stained (blue) and unstained (pale yellow) cells were counted under inverted microscope. The relative THb production was expressed as (number of stained cells/total number of cells) × 100%. The quantitative amount of Hb was determined by using the plasma Hb kit (Sigma) according to the manufacturer's instruction.

### 2.5. HbF Assay

HbF levels of K562 cells were determined quantitatively by commercially available enzyme linked immunosorbent assay (ELISA) kit (Bethyl). At each time interval, diluted cell lysate (in 50 mM Tris-Cl/1% BSA) was transferred to 96-well plate, which was pre-coated with anti-HbF, and incubated at room temperature for 1 h. The secondary antibody, horse radish peroxidase, was conjugated to primary antibody for 1 h followed by the addition of 10% TMB solution. The reaction was terminated by adding 2 M H_2_SO_4_ and the absorbance was determined at 450 nm. The concentration of HbF was determined in reference to the standard curve and expressed as HbF per total proteins.

### 2.6. Quantitative Real-Time Polymerase Chain Reaction on *α*-and *γ*-Globin mRNA

The cells were seeded onto six-well plate at a density of 1 × 10^4^ cells mL^−1^ and incubated with corresponding FT-EtOH extract at various concentrations and HU for 6 days at 37°C. After 6 days treatment, total RNA was isolated from cultures of each experimental group using RNeasy mini kit according to manufacturer's instruction (Qiagen). Eighty nanograms of total RNA extracted from each treatment group were subjected to one-step real-time reverse transcription polymerase chain reaction (RT-PCR) using the QuantiFast SYBR Green RT-PCR kit (Qiagen) with ABI 7500 Fast Real-Time PCR System. *α*- and *γ*-globin gene-specific primers were design customized and validated by manufacturer (Hs_HBA1_2_SG, QT01671215; Hs_HBG2_1_SG, QT00040068; QuantiTect Primer Assays; QIAGEN). Each reaction mix contained the following: 80 ng RNA, I *μ*M of each primer, 12.5 *μ*L 2 × QuantiTect SYBR green RT-PCR master mix, 0.25 *μ*L QuantiTect reverse transcriptase mix and nuclease-free H_2_O to 25 *μ*L. The following thermal cycling conditions were used: for reverse transcription, 10 min at 50°C; for the PCR initial activation step, 5 min at 95°C; for amplification, 15 s at 95°C, 30 s at 55°C for 45 cycles. Melting curve analysis of the RT-PCR products was done in order to verify the specificity and identity of the PCR products. For each sample, real-time RT-PCR analysis was done with a reference gene (glyceraldehyde-3-phosphate dehydrogenase gene, GAPDH) as an internal control.

Relative gene expression levels are presented as 2^(−ΔCt)^, where ΔCt = Ct_target_  −  Ct_GAPDH_ and the Ct value is the cycle threshold. The SD was determined from three independent experiments of the ΔCt values. The upper and lower errors were defined as 2^−(ΔCt−SD)^ and 2^−(ΔCt+D)^, respectively. Fold change for the treatment group was defined as the relative expression, compared with the control group without treatment and was calculated as 2^(−ΔΔCt)^, where ΔΔCt_treatment_ = ΔCt_treatment_ – ΔCt_control_.

### 2.7. Western Blotting Analysis

Treated K562 cells were lysed in 200 *μ*L of ice-cold lysis buffer (50 mM Tris, 150 mM NaCl, 2 mM EDTA, 50 mM NaF, 0.2 mM sodium vanadate, 1% SDS, 1 mM phenylmethylsulphonyl fluoride, 1 *μ*g mL^−1^ aprotinin and 1 *μ*g mL^−1^ leupeptin), and protein concentrations were determined using bicinchoninic acid assay. Cytosolic extracts (20 *μ*g) were subjected to SDS-polyacrylamide gel electrophoresis using 10% polyacrylamide gels and transferred to polyvinylidene *ﬂ*uoride membranes (Biorad). Membranes were blocked with Tris-buffered saline containing 0.1% Tween-20 and 10% dry milk for 1 h and then incubated overnight at room temperature with diluted primary antibodies (anti-ERK1/2 phosphorylated, anti-ERK1/2 total, anti-stress-activated protein kinase/c-Jun NH_2_-terminal kinase (SAPK) phosphorylated, anti-SAPK total, anti-p38 phosphorylated and anti-p38 total antibodies; Calbiochem). After incubation, the membranes were washed with TBS-T followed by incubation for 1 h with secondary antibodies conjugated horseradish peroxide diluted at 1 : 3000. The proteins of interest were visualized by the chemiluminent protein detection system according to the manufacturer's instructions (Biorad).

### 2.8. Statistical Analysis

All data are expressed as mean  ±  standard deviation (SD). All the statistical analysis were performed by software SigmaStat version 3.5 (SPSS Science Software GmbH, Erkrath, Germany). Statistical significance was compared between each treatment groups and control group by one-way ANOVA followed by *post-hoc* Dunnett test. Values with *P* < .05 were considered as statistically significant. Values of IC50 and EC50 were determined by non-linear regression dose-response curve.

## 3. Results

### 3.1. Cytotoxicity Assay

**Figure 1:**
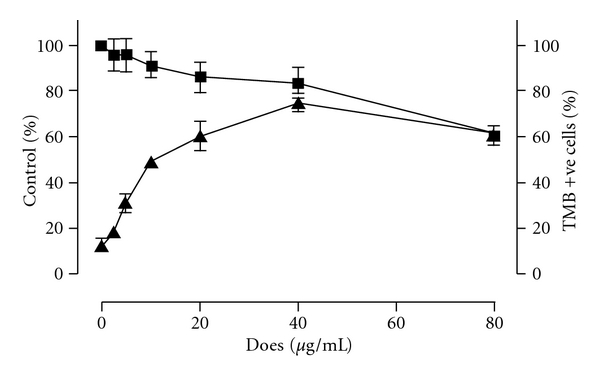
The effect of FT-EtOH extract on the cell viability and THb induction in K562 cells. (i) K562 cells were treated with indicated concentrations of FT-EtOH extract for 2 days. The cell viability (filled square) was then estimated by MTT assay. (ii) K562 cells were treated with indicated concentrations of FT extract for 6 days. The Hb-positive cells were stained by TMB assay and the percentage of stained cells (filled triangle) was calculated. The results are expressed as mean  ±  SD of three cultures. The data are representative of three separate experiments. ^#^ *P* < .05, significant difference in cell viability when compared with non-treated group. *P < .05, significant difference in the number of TMB-stained cells when compared with non-treated group.

The cytotoxic effect of FT-EtOH extract on K562 cells is shown in Figure 1. There was a dose-dependent inhibitory effect of FT-EtOH extract. Significant cytotoxic effect was found when the cells were treated with ≥80 *μ*g mL^−1^ extract. The IC50 of the FT-EtOH extract was 108.6 *μ*g mL^−1^.

### 3.2. THb Assay

Our results demonstrated that the FT-EtOH extract induced THb formation in the range without cytotoxic effects. At 40 *μ*g mL^−1^, the extract exerted its maximal effect on THb production by 78.0  ±  4.2% (Figure 1). Beyond this concentration, the degree of Hb induction was decreased, possibly due to increasing cytotoxic effect as found in MTT assay.

**Figure 2:**
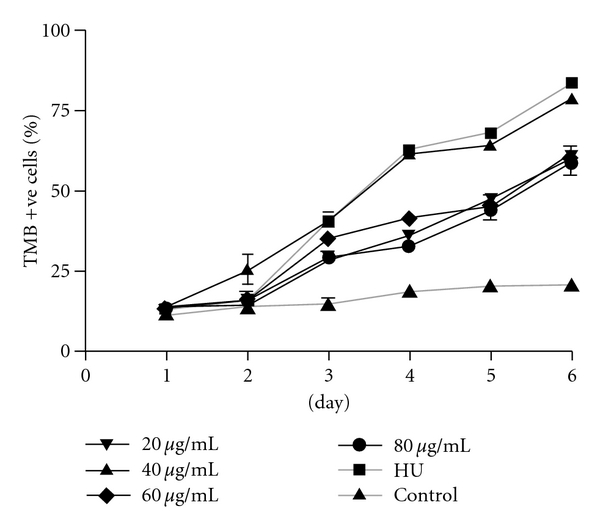
The effect of FT-EtOH extract and HU (25 *μ*g mL^−1^) on induction of THb of K562 cells at different time intervals. K562 cells were treated with indicated concentrations of FT extract and HU for 6 days. The Hb-positive cells were stained by TMB assay. The results are expressed as mean  ±  SD of three cultures. The data are representative of three separate experiments.

Time-dependent response of THb-induction effect of FT-EtOH extract is shown in Figure 2. From day 3 to day 6, there was a significant increase of TMB-positive cell percentage at all test concentrations of FT extract when compared with their respective basal values on day 1. Our data showed that the optimal dosage of this extract was 40 *μ*g mL^−1^, which gave similar time-dependent Hb-inducing effect as positive control HU at 25 *μ*g mL^−1^. In the control group, the percentage of TMB-positive cells was slightly increased (<15%). It indicated that auto-differentiation of K562 cells was found in prolong culture.

### 3.3. HbF Assay

**Figure 3:**
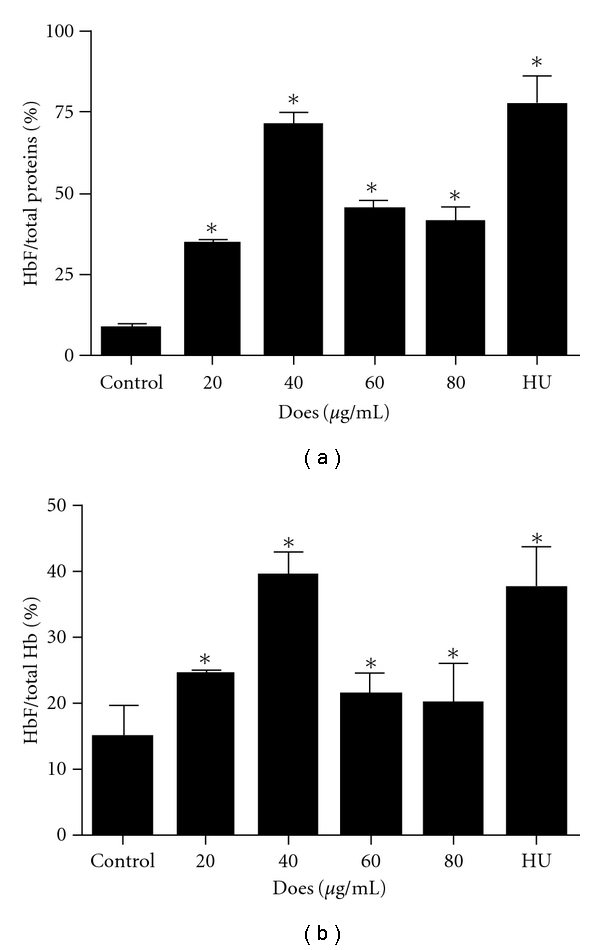
The effect of FT-EtOH extract and HU (25 *μ*g mL^−1^) on HbF production of K562 cells. Dose-dependent HbF-inducing effects of FT-EtOH extract on K562 cells after 6 days of treatment, expressed in (a) percentage of HbF per total proteins and (b) percentage of HbF per THb. The HbF concentration of each group was quantified by commercial ELISA kit. The results are expressed as mean  ±  SD of three cultures. The data are representative of three separate experiments. *P < .05, significantly different from non-treated control group.

There was a significant increase of HbF at all tested concentrations (20–80 *μ*g mL^−1^) of FT-EtOH extract when compared with untreated cell control [Figure 3(a)). Dose-dependent response of FT-EtOH extract was observed in the range of 20–40 *μ*g mL^−1^. The maximal response was observed at 40 *μ*g mL^−1^. Beyond this dose, the HbF-inducing effect of FT was diminished, possibly due to its cytotoxic effects at higher dose. Time-dependent effect of FT-EtOH extract was also demonstrated during 6 days of incubation (data not shown). Similar HbF-inducing effect was demonstrated in HU (25 *μ*g mL^−1^) group, it increased the HbF concentration significantly over 6 days of culture period. During the experiment, auto-differentiation of K562 cells was found in the untreated control cells starting from day 4 to day 6 (data not shown). However, the degree of increase was smaller than both treatment and positive control groups.

To investigate the specificity of FT-EtOH extract on HbF stimulation, we determined the HbF to THb ratio. FT-EtOH extract increased the proportion of HbF relative to total K562 cells. At 40 *μ*g mL^−1^, FT-EtOH extract caused a 2.6-fold increase of the HbF ratio compared with untreated control cells, its HbF-inducing effects were similar to the positive control HU.

### 3.4. Quantitative Real-Time PCR on *α*- and *γ*-Globin mRNA

**Figure 4:**
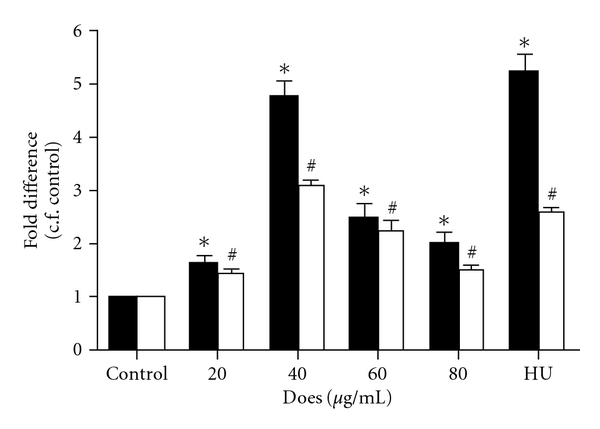
The effect of FT-EtOH extract and HU (25 *μ*g mL^−1^) on *α*- (filled square) and *γ*-globin (open square) expressions of K562 cells. K562 cells were treated with indicated concentrations of FT-EtOH extract and HU for 6 days. The mRNA expression of *α*- and *γ*-globin gene of each group was quantified by real-time PCR. The results are expressed as mean  ±  SD of three cultures. The data are representative of three separate experiments. ^∗,#^ *P* < .05, significantly different from corresponding non-treated control group.

Following 6 days of treatment, *γ*-globin mRNA level was increased significantly at all investigated concentrations (20–80 *μ*g mL^−1^) of FT-EtOH extract when compared with untreated cell control (Figure 4). This extract showed a dose-dependent effect on *γ*-globin mRNA induction in the range of 20–40 *μ*g mL^−1^. At 40 *μ*g mL^−1^, the mRNA level of *γ*-globin was increased 4.8  ±  0.5 fold compared with untreated control cells. The induction effect of FT-EtOH extract on *γ*-globin mRNA expression was diminished at higher concentrations. Similar response was observed in HU (25 *μ*g mL^−1^) group that showed 5.2  ±  0.6 fold increase. The mRNA expression of *α*-globin was also increased, but to a lesser extent. At 40 *μ*g mL^−1^, the mRNA level of *α*-globin was increased by 3-fold. Our data implied that FT-EtOH extract exhibits relative specificity for *γ*-globin mRNA expression, as suggested by the protein data.

### 3.5. Inhibitory Effects of Specific MAPK Inhibitors on FT-Treated Cultures

**Figure 5:**
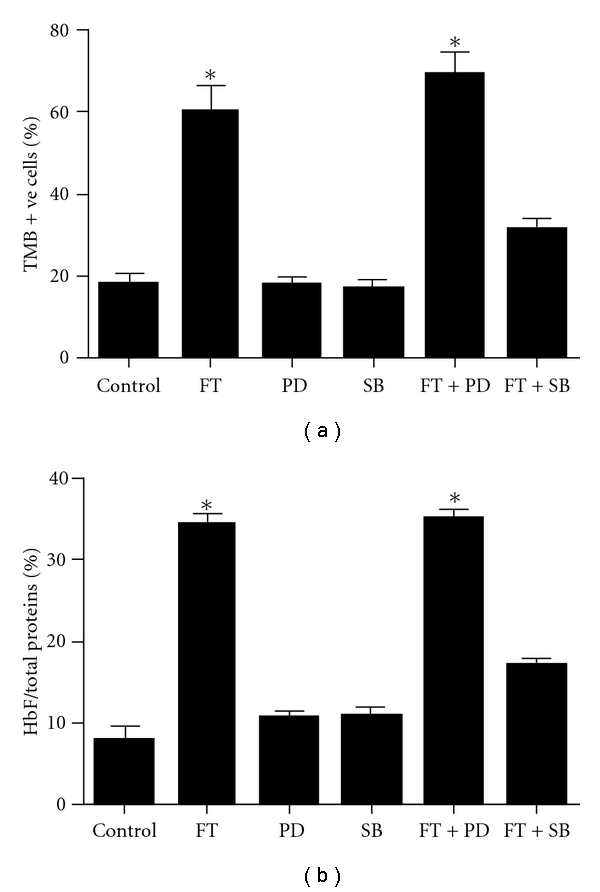
The effect of MAPK inhibitors on FT-induced total HbF expression in K562 cells. Cells were cultured in the presence of 20 *μ*g mL^−1^ FT-EtOH extract with addition of ERK-inhibitor PD98059 (PD) and p38-inhibitor SB203580 (SB), respectively. (a) The number of TMB-stained cells and unstained cells was scored under inverted microscope at 200x magnification. The relative THb production of K562 cells was expressed as a percentage of number of stained cells in reference to total number of cells. (b) The concentration of HbF was determined in reference to the standard curve of HbF and expressed in percentage of HbF per total proteins. The results are expressed as mean  ±  SD of three cultures. The data are representative of three separate experiments.

To determine whether MAPK-mediated signaling pathways are involved in the FT-driven HbF induction, the actions of ERK inhibitor PD98059 and p38 inhibitor SB203580 in FT-treated K562 cultures were examined. As shown in Figure 5(a), SB203580 significantly inhibited the FT-induced Hb production completely by 48%, whereas PD98059 did not pose any significant effects on FT-treated culture. Similarly, p38 inhibitor SB203580 also significantly decreased FT-induced HbF synthesis (Figure 5(b)). On day 6, the percentage of HbF/total proteins of FT plus SB group was 17.2  ±  0.7, which was significantly lower than FT group by 50%. On the other hand, the ERK inhibitor PD98059 did not alter the production of HbF significantly (*P* > .05) when compared with FT group.

### 3.6. Western Blotting Analysis on Signaling Pathways

**Figure 6:**
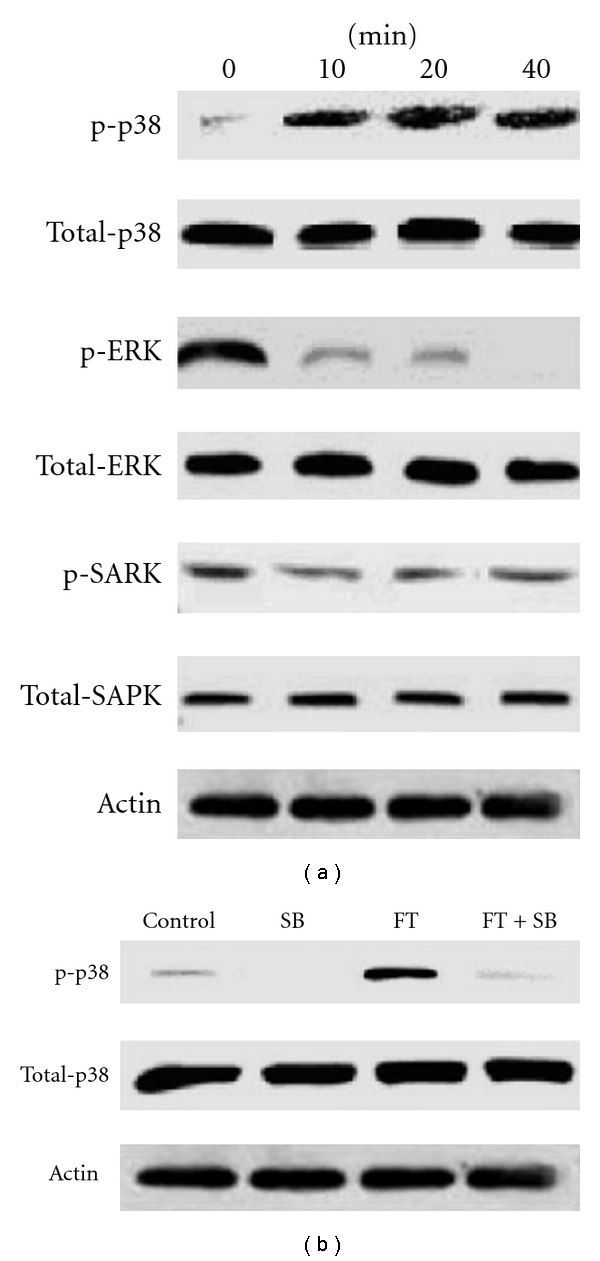
The effect of FT-EtOH extract on MAPK-mediated signaling pathways. (a) K562 cells were treated with FT extract for 10 min. Cells were lysed and analyzed by western blotting with antibodies probing the phosphor- and total forms of p38, ERK and SAPK. (b) K562 cells pre-treated with p38-inhibitor SB203580 (SB) for 10 min in the presence of FT-EtOH extract for 10 min. Cells were lysed and analyzed by western blotting. Similar results were obtained in three separate experiments.

The p38, ERK and SAPK pathways are thought to be involved in HbF activation. We tested whether FT affected these pathways on K562 cells. As shown in Figure 6(a), noticeable basal levels of p-ERK and p-SAPK were found, since both the proteins were actively involved in cell proliferation and survival at undifferentiated state [15, 16]. For p38, it was substantially phosphorylated after 10 min of FT-EtOH extract addition and this phosphorylation was sustained for the entire observation period. In contrast, ERK was de-phosphorylated upon FT treatment in time-dependent manner. However, no significant change was found in the phosphorylation of SAPK protein. In addition, the treatment of p38 inhibitor SB203580 down-regulated the FT-induced phosphorylation of p38 (Figure 6(b)). These results indicate that activation of p38 is essential for FTinduced HbF production.

## 4. Discussion

To date, the use of extracts from medicinal plants for biomedical purposes is increasing for the treatment of various diseases such as diabetes mellitus [17], cardiovascular diseases [18] and bone disorders [19]. However, for the case of *β*-hemoglobinpathies, only few examples are available. For instance, Niprisan (Nix-0699), a ethanol/water extract from Nigeria indigenous plants, demonstrated a significant anti-sickling effects *in vitro* and *in vivo* [20, 21]. Recently, bergamot fruit extracts was recently reported as a good *γ*-globin gene expression inducer in both K562 and human progenitor cells [22]. The extract of Ayurvedic herb, *Aegle Marmelos* was also found to be associated with the activation of erythroid differentiation and HbF induction in K562 human erythroleukemia cells [23]. In addition, phyotochemicals (i.e., angelicin and resveratrol) were shown to be potent inducers of erythroid differentiation and gamma-globin gene expression in either K562 or human erythroid progenitor cells [24, 25].

**Figure 7:**
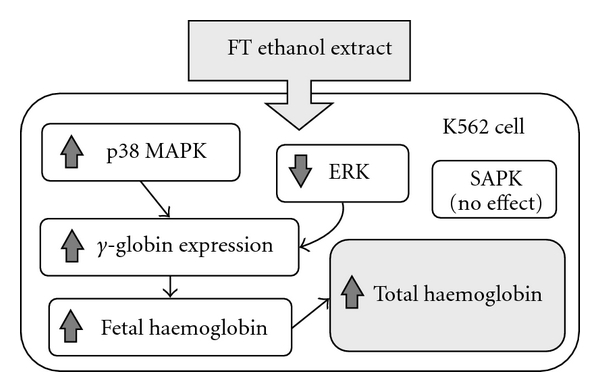
Hypothetical diagram to illustrate the actions of FT-EtOH on MAPK family in HbF induction. After FT-EtOH treatment, activation of p38 and inhibition of ERK lead to HbF production and subsequently increase total hb level in K562 cell. However, SAPK does not involve in FT-EtOH-induced erythroid differentiation process.

In the present study, we found that the ethanol extract of FT, like HU, significantly increased the production of HbF in K562 cells. This was indicated by the increase of HbF protein levels and *γ*-globin mRNA synthesis, following the treatment with FT extract. THb levels were also elevated. We found that the activation of HbF synthesis involved the inhibition of ERK and activation of p38 MAPK signaling pathways as shown in western-blotting analysis. Specific inhibition of p38 signaling by SB203580 inhibited FT-EtOH-mediated induction of Hb production.  Figure 7 shows a schematic drawing summarizing the experimental results.

Our results demonstrated that FT-EtOH extract is effective in increasing *γ*-globin mRNA synthesis and HbF production in the concentrations without killing the cells. FT is long regarded as non-toxic because it has been used in China over thousands of years with no severe adverse side-effects. This extract is relatively safe for long-term treatment, when compared with HbF-inducing chemotherapeutic agents. In addition, it was recently reported that accelerated apoptosis of erythroid progenitors in *β*-thalassemia is a major obstacle of definitive therapy, since most of the HbF-inducing agents are cytotoxic that inhibits the cell proliferation and cause cell growth arrest [26]. As a result, the beneficial effects of these agents on globin chain balance may not be inducible in apoptotic cells. Because of this, agents with low cytotoxicity, such as FT-EtOH extract, may decrease the cellular apoptosis of *β*-thalassaemic erythroid cells and provide more favorable conditions for effective erythropoiesis.

We studied three signaling cascades related to erythroid differentiation, including p38 MAPK, ERK and SAPK. It has been reported that HU mediates erythroid differentiation and HbF production by inhibition of ERK and activation of p38 MAPK signal transduction pathways [27]. Our results were in line with those reports, suggesting that the effects of FT-EtOH extract on erythroid differentiation in K562 cells share some similarities. In K562 cells, activation of ERK signaling leads to megakaryocytic differentiation, while inhibition of ERK pathway enhances the erythroid phenotype and also the *γ*-globin mRNA expression [28]. In addition, ERK pathway inhibitor U0126 was reported to induce *γ*-globin expression and HbF formation in human erythroid progenitor cells [29]. Recently, Mabaera *et al.* suggested that p38 MAPK plays a central role in HbF re-activation [30]. p38 MAPK signal pathway is activated by various environmental stresses and has been suggested to play a critical role in apoptosis, cell growth and erythroid differentiation. There has been accumulating evidence from K562 cells and human progenitor erythroid cells on the involvement of p38 MAPK up-regulation in HbF-induction process [31, 32]. Furthermore, p38 MAPK signaling pathway has been implicated in the actions of several HbF-inducing agents, including butyrate [33], apicidin [34] and trichostatin A [35]. Our present study suggests that FT-EtOH extract induced erythroid differentiation and HbF production by inhibiting ERK and activating p38 MAPK signaling transduction. However, the action mechanism for the activation or inactivation of MAPKs is still not clear. It was proposed that multiple upstream signals, including DNA damage, oxidative stress, heat shock, osmotic shock, nitric oxide and inhibition of protein synthesis would activate the downstream kinases and transcription factors for HbF production [30].

Recently, there were conflicting reports concerning the involvement of SAPK in erythroid differentiation [27, 32, 33, 36]. Activation of SAPK was shown to be essential for erythropoietin-induced erythroid differentiation of mouse erythroleukemia cells [32]. However, a significant inhibition of SAPK was found when the K562 cells were treated with HU [27]. Apart from these findings, short chain fatty acid derivatives (SCFAD), such as butyrate and valproate, did not cause any effect on SAPK pathway in K562 cells [33, 36]. Therefore, the role of SAPK in HbF induction still remains to be elucidated. In our present study, FT-EtOH extract did not cause SAPK phosphorylation and it was found to induce a similar pattern of MAPK modulation as SCFAD, suggesting that both of them have intracellular signaling pathways in common.

The major limitation of our study is that FT-EtOH extract contains numerous compounds. Their respective pharmacokinetics and biotransformation properties *in vivo* are not well-defined. The active compounds may be metabolized before they exert direct effects on erythroid progenitor cells, especially for those orally administrated therapeutics. In addition, FT-EtOH extract may contain other substances that interfere with the biological actions of the active ingredients *in vivo*. Therefore, development of *β*-thalassemia animal model is important for accurately predicting the therapeutic effects *in vivo*.

In summary, our results present the first evidence on the HbF-inducing actions of ethanol extract of FT on K562 cells as demonstrated by the elevation of *γ*-globin mRNA expression and HbF levels. The induction effects of FT-EtOH extract might be mediated by its direct action of stimulating p38 MAPK and inhibiting ERK signaling pathways. Our observations would serve as groundwork for further studies. In order to develop FT-EtOH extract in the international scientific community as an alternative regime for the treatment of *β*-thalassemia by HbF stimulation, additional speculation awaits the illustration of the effect of FT-EtOH extract on primary precursor cells from normal donors or *β*-thalassaemic patients. And also additional experiments will be required to perform acute and chronic toxicity test *in vivo* to ensure the extract is safe for clinical use on human subject. Finally, the efficacy of the extract will be examined in clinical evaluation. It is anticipated that FT-EtOH extract is a promising alternative and complementary agent for the management of *β*-thalassemia.

## Funding

The Ming Lai Foundation and Hong Kong Innovation and Technology Fund UIM/168.
